# Cytokine Diversity in the Th1-Dominated Human Anti-Influenza Response Caused by Variable Cytokine Expression by Th1 Cells, and a Minor Population of Uncommitted IL-2+IFNγ- Thpp Cells

**DOI:** 10.1371/journal.pone.0095986

**Published:** 2014-05-01

**Authors:** Nan Deng, Jason M. Weaver, Tim R. Mosmann

**Affiliations:** David H. Smith Center for Vaccine Biology and Immunology, University of Rochester Medical Center, Rochester, New York, United States of America; St. Jude Children's Research Hospital, United States of America

## Abstract

Within overall Th1-like human memory T cell responses, individual T cells may express only some of the characteristic Th1 cytokines when reactivated. In the Th1-oriented memory response to influenza, we have tested the contributions of two potential mechanisms for this diversity: variable expression of cytokines by a uniform population during activation, or different stable subsets that consistently expressed subsets of the Th1 cytokine pattern. To test for short-term variability, *in vitro*-stimulated influenza-specific human memory CD4+ T cells were sorted according to IL-2 and IFNγ expression, cultured briefly *in vitro*, and cytokine patterns measured after restimulation. Cells that were initially IFNγ+ and either IL-2+ or IL-2- converged rapidly, containing similar proportions of IL-2-IFNγ+ and IL-2+IFNγ+ cells after culture and restimulation. Both phenotypes expressed Tbet, and similar patterns of mRNA. Thus variability of IL-2 expression in IFNγ+ cells appeared to be regulated more by short-term variability than by stable differentiated subsets. In contrast, heterogeneous expression of IFNγ in IL-2+ influenza-specific T cells appeared to be due partly to stable T cell subsets. After sorting, culture and restimulation, influenza-specific IL-2+IFNγ- and IL-2+IFNγ+ cells maintained significantly biased ratios of IFNγ+ and IFNγ- cells. IL-2+IFNγ- cells included both Tbet^lo^ and Tbet^hi^ cells, and showed more mRNA expression differences with either of the IFNγ+ populations. To test whether IL-2+IFNγ-Tbet^lo^ cells were Thpp cells (primed but uncommitted memory cells, predominant in responses to protein vaccines), influenza-specific IL-2+IFNγ- and IL-2+IFNγ+ T cells were sorted and cultured in Th1- or Th2-generating conditions. Both cell types yielded IFNγ-secreting cells in Th1 conditions, but only IL-2+IFNγ- cells were able to differentiate into IL-4-producing cells. Thus expression of IL-2 in the anti-influenza response may be regulated mainly by short term variability, whereas different T cell subsets, Th1 and Thpp, may contribute to variability in IFNγ expression.

## Introduction

The major immune mechanism of protective immunity to influenza virus is often considered to be neutralizing antibody, partly because the seasonal variation in influenza strains involves mainly antibody epitopes. However, CD4 T cells may also play an important role in immunity to influenza [1]–[5]. CD4 cells could provide help to induce antibody production by B cells, induce effector CD8 T cells, and protect the host by helper-independent mechanisms [4], [6]. T cell responses may also be significant as a cause of immunopathogenesis [4], [7]. Influenza-specific human CD4 T cell memory responses are biased strongly towards Th1 cells, producing TNFα, IFNγ and IL-2 on activation. IFNγ contributes indirectly and directly to protection, inhibits the Th17 response and can cause immunopathogenesis [4]. However, not all influenza-specific CD4 T cells express both IL-2 and IFNγ.

Within an overall Th1 response, individual cells may express subsets of the Th1 cytokine pattern [8]. “Multifunctional” T cells produce a greater number of cytokines, and an increased proportion of multifunctional cells correlates with better protection against Leishmania major in mice [9], slower progression of HIV infection to AIDS [10], and protection by vaccines against yellow fever [11]–[13], and vaccinia virus [14]. However, in tuberculosis [15], multifunctional T cells correlate with increased disease.

Diverse cytokine patterns can be explained by either variable expression of Th1 cytokines by *bona fide* Th1 cells, or multiple T cell differentiation phenotypes, or a combination of these two possibilities. Expression of some cytokine genes appears to be regulated by a stochastic or probabilistic mechanism, for example IL-4 in a pure Th2 population [16], or IL-2 and IFNγ in a Th1 population [8]. Stochastic expression of IL-4 and IL-2 could be due to the same mechanism that causes mono-allelic expression of IL-4 [17], [18] and IL-2 [19]. In humans, the Th2 cytokines IL-4 and IL-5 are often expressed by different cells if memory cells are stimulated directly *ex vivo*, but are expressed simultaneously by many cells after *in vitro* culture [20],(Y. Huang, and T.R. Mosmann, unpublished). Less is known about variable IL-2 and IFNγ expression in human memory cells. The stochastic model could explain preferential multi-producer or single-producer responses, if it is assumed that different immune responses alter the probability of stochastic expression.

Variability of cytokine expression could also be explained by a combination of two or more different T cell phenotypes, in which the different cytokine patterns are expressed by cells in stable states of differentiation, such as primed T helper cell precursors (Thpp), which express IL-2 but not effector cytokines such as IL-4, IFNγ or IL-17 [21], [22]. These Thpp cells are uncommitted with respect to further effector cell differentiation, as single Thpp cells can differentiate into either Th1 or Th2 T cells [21]–[23]. This cell population overlaps partially with the CD4 central memory population (Tcm) although the two types are not synonymous [24], [25]. Human responses to protein vaccines, such as tetanus, diphtheria and HBV, are Thpp dominated. In contrast, the response to infections by influenza (and other viruses) is strongly Th1-biased [22]. This IFNγ+ bias is particularly clear in the response to long-circulating influenza strains, whereas a new pandemic influenza strain induced a mixed influenza-specific response [24] including both IL-2+IFNγ- and IL-2+IFNγ+ cells (abbreviated 2+γ- and 2+γ+, respectively). Similarly, the 2-γ+ cytokine expression pattern may be due to a population of exhausted Th1 cells [26]–[28] such as those expressing PD-1 and Tim3 [29], [30].

To distinguish the relative contributions of short-term versus pre-determined variability of Th1 cytokine expression in influenza responses, we used a combination of sorting, restimulation, evaluation of Tbet expression, RNAseq and *in vitro* differentiation to show that both mechanisms appeared to operate in influenza-specific or polyclonally-activated human memory CD4 T cells. The 2-γ+ and 2+γ+ phenotypes appeared to be in short-term equilibrium, whereas 2+γ- cells included uncommitted Thpp-like cells that were stable in the short term, but could subsequently differentiate into either IFNγ-producing or IL-4-producing phenotypes under appropriate conditions.

## Materials and Methods

### Ethics Statement

All procedures were approved by the Research Subjects Review Board at the University of Rochester Medical Center, Rochester, New York. Participants provided written, informed consent to participate in the study. The consent procedure was approved by the Research Subjects Review Board.

### Human sample collection

Peripheral blood samples were collected into heparinized vacutainer tubes from healthy adult donors. Ficoll-hypaque (Cellgro, Herndon, VA) gradient centrifugation was used to isolate peripheral blood mononuclear cells (PBMC). The layer of lymphocytes was collected and washed with R8 medium (8% FBS in RPMI1640) and cryopreserved in freezing buffer (90% FBS, 10% DMSO).

### Antibodies

Anti-human antibodies are listed in Table 1.

**Table 1:** Fluorescent antibody conjugates.

Antigen	Fluorochrome	Clone	Supplier	Sorting or analysis (S, A)?
TNFa	Pacific Blue	MP9-20A4	Biolegend	A
Live/Dead	Yellow	N/A	Invitrogen	A
CD3	Qdot605	UCHT1	Invitrogen	A
CD45RA	Qdot655	MEM-56	Invitrogen	A, S
CD8	Qdot705	3B5	Invitrogen	A
CD14	Qdot800	TuK4	Invitrogen	A, S
IL-2	AF700	MQ1-17H12	Biolegend	A
CD69	APC Cy7	FN-50	BD	A
Tbet	PE	4B10	eBioscience	A
CD4	PE TR	S3.5	Invitrogen	A, S
IFNγ	PE Cy7	B27	Biolegend	A
IFNγ	FITC	N/A	miltenyi	S
TNFa	APC	N/A	miltenyi	S
CD56	AF700	HCD56	Biolegend	S
CD154	APC Cy7	24-31	Biolegend	S
IL-2	PE	N/A	miltenyi	S
CD19	PE Cy5	HIB19	Biolegend	S
CD8	PE Cy7	3B5	eBioscience	S
				

### Antigens

The influenza A/CA/09 ‘very different’ peptide pool (Vdiff) comprised selected Influenza A/California/04/09 peptides (unique with respect to two other H1N1 strains, A/New Caledonia/20/99 and A/Brisbane/59/07) with 15–17 amino acid residues, offset by 5 amino acids [24]. The Vdiff peptide pool does not stimulate significant responses in pre-pandemic PBMC samples [24] and so the responses seen in this study (using post-pandemic PBMC samples) were almost certainly primed by infection or vaccination with the CA/09 pandemic strain. The Tetanus peptide pool comprised CD4 T cell-restricted epitopes [31]: L31–50, L271–290, L286–305, H56–75, H116–135, H131–150, H161–180, H176–195, H191–210, H251–270, H373–387, H431–450, H491–510, H566–585, H731–750, H791–810; where L and H are Light and Heavy chains, respectively (synthesized by Mimotopes, Clayton, Australia). Influenza and tetanus peptide pools were used at final concentrations of 0.1 µg/ml/peptide and 3 µg/ml/peptide, respectively. TIV for 2011 contained A/California/7/2009 (H1N1), A/Perth/16/2009 (H3N2) and B/Brisbane/60/2008 (FluLaval, GlaxoSmithKline), and was used at a final concentration of 1 µg/ml. Staphylococcus Enterotoxin B (SEB, Sigma) was used at a final concentration of 1 µg/ml.

### Stimulation of Human PBMCs

PBMC were thawed and cultured in R8 medium overnight at 37°C and 5% CO2. Viable cell counts were determined by trypan blue staining. 1–2*10^6^ cells were plated into each well of 96-well round-bottom plates. The seasonal influenza trivalent inactivated vaccine (TIV), the A/CA/09 peptide pool or the Tetanus peptide pool were added to a total of 200 µl R8 medium. Plates were cultured at 37°C and 5% CO2. After two hours, 3 µg/ml Brefeldin A and 2.0 µM monensin were added, and after another 6 hours stimulation, cells were harvested and stained.

### Intracellular cytokine and transcription factor staining

Cells were collected and washed with wash buffer. Surface markers were stained in wash buffer on ice for 80 minutes. Then the cells were permeabilized and fixed for 30 minutes in intracellular staining perm/fix buffer (Invitrogen, Grand Island, NY). After washing twice with intracellular staining perm buffer, cytokines were stained in the perm buffer on ice for 80 minutes. If Tbet staining was not needed, cells were washed and analyzed. For Tbet staining, the cells were further permeabilized and fixed in Transcription Factor Staining perm/fix buffer (eBioscience) for 20 minutes. After washing twice with the same buffer, Tbet was stained in the perm buffer on ice for 80 minutes. Then cells were washed and the staining signals were measured by an LSRII cytometer (BD Immunocytometry Systems, San Diego, CA) and analyzed using FlowJo software (Treestar, San Carlos, CA).

### Cell sorting of cytokine-expressing cells

Human PBMCs were stimulated with TIV (2011 vaccine year) or SEB for 4 hours. After collection, the cells were stained using the Cytokine Secretion Assay for IL-2, IFNγ and TNFα according to the manufacturers recommendations (Miltenyi, Auburn, CA). The 2+γ+, 2+γ-, 2-γ+ and 2-γ- cells in activated human PBMCs (CD4+CD8- CD14+CD56- CD19- CD45RA- CD69+TNFα+) were sorted by a FACSAria-II cell sorter into R8 medium.

### Culture of sorted T cells and secondary stimulation

Human 2+γ+, 2+γ-, 2-γ+ and 2-γ- cells were washed with R8 medium and plated into 96 well plates. Each well contained <500 sorted cells in 200 µl R8 supplemented with 1 ng/ml human IL-2 (eBioscience), 10 µg/ml anti-IL4 antibody (MP4-25D2, eBioscience), 10 µg/ml anti-IL-12 antibody (C8.6, eBioscience) and 10 µg/ml anti-IFNγ antibody (NIB42, eBioscience). 36 or 108 hours later, the cells were restimulated with PMA (25 ng/mL) and Ionomycin (1 µg/mL) (P/I) and stained by the ICS assay. Polyclonal P/I stimulation was used because antigen or SEB specificity was established by sorting, and P/I was a more efficient stimulus.

### Real-time PCR (RT-PCR)

Cells were stimulated with PMA and ionomycin (to avoid a contribution by APC to mRNA levels) for 6 hours. The RNA was extracted by Qiagen RNA microprep kit (Qiagen) and reverse transcribed into cDNA using the Superscript III First Strand DNA Synthesis kit (Invitrogen, Grand Island, NY). Expression levels of IL-2, IFNγ, IL-4 and CD3ε were measured by RT-PCR using Taqman gene expression assays (Life Technologies). The expression levels of IL-2, IFNγ and IL-4 were normalized to the expression level of CD3ε.

### RNA-Seq

T cells expressing the 2+γ+, 2+γ- and 2-γ+ patterns were sorted directly into lysis buffer for RNA extraction. RNA samples were amplified and sequenced following Linnarsson's protocol [32] in the Functional Genomics Center of the University of Rochester Medical Center. Significant genes (P<0.001) were determined by the DEGseq package [33].

### Generation of monocyte-derived DC (moDC) from human PBMC

PBMCs were plated in 12 mm cells dishes at a density of 2*10^6^ cells/ml in X-VIVO 15 serum-free medium (Biowhittaker, Walkersville, MD, USA) supplemented with Antibiotic-Antimycotic (Gibco, Grand Island, NY). Cells were incubated at 37°C and 5% CO_2_ for 90 minutes for monocyte adherence then the non-adherent cells were removed by washing twice with X-VIVO medium and then once with cold PBS. The adherent cells were cultured in X-VIVO medium containing 500 IU/ml GM-CSF (eBioscience) and 500 IU/ml IL-4 (eBioscience). On days 3 and 5, fresh GM-CSF/IL-4 medium was added. On Day 6, TNFα (eBioscience) was added at a final concentration of 50 ng/mL to induce DC maturation. Mature DCs were harvested on day 8. They were loaded with influenza antigen and irradiated at 10000Rad before usage.

### Redifferentiation of sorted human influenza-specific T cells

Human 2+γ+, 2+γ-, 2-γ+ and 2-γ- CD4 T cells were sorted into R8 medium, washed in R8 medium and plated into a 96 well plate. Each well contained <100 sorted cells and 500 moDCs in 200 µl R8 with Th1- or Th2-inducing conditions. The Th1 cultures contained 1 ng/ml IL-2 (eBioscience), 1 ng/ml IL-12 (eBioscience) and 10 µg/ml anti-IL4 antibody. Th2 cultures contained 1 ng/ml IL-2, 10 ng/ml IL-4, 10 µg/ml anti-IL-12 and 10 µg/ml anti-IFNγ. Half of the medium was changed every 2 days with the same medium. After 2 weeks, the cells were restimulated with PMA and Ionomycin and analyzed by RT-PCR.

## Results

In preliminary experiments, *in vitro*-generated mouse allospecific Th1 cells were restimulated and analyzed by ICS and flow cytometry. These cells uniformly expressed the Th1 regulator Tbet, but expressed all combinations of IL-2 and IFNγ (data not shown), consistent with previous studies [8]. Sorting and restimulation of the 2+γ+, 2+γ-, 2-γ+ and 2-γ- populations showed that, regardless of the original cytokine pattern, all four populations contained similar proportions of IFNγ+ and IFNγ- cells. This rapid convergence of the cytokine patterns suggested that in *in vitro* Th1 cell lines, different cytokine expression patterns were due to short-term random effects. A kinetic analysis using the two-color Fluorospot assay [34], [35] for IL-2 and IFNγ showed that the 2+γ+, 2+γ- and 2-γ+ cells stably expressed these patterns over at least 48 hours (data not shown), indicating that the variable patterns were not due to the short time window of the ICS assay.

### Stable and rapidly-randomizing cytokine patterns in human memory Th1-like cells

**Figure 1:**
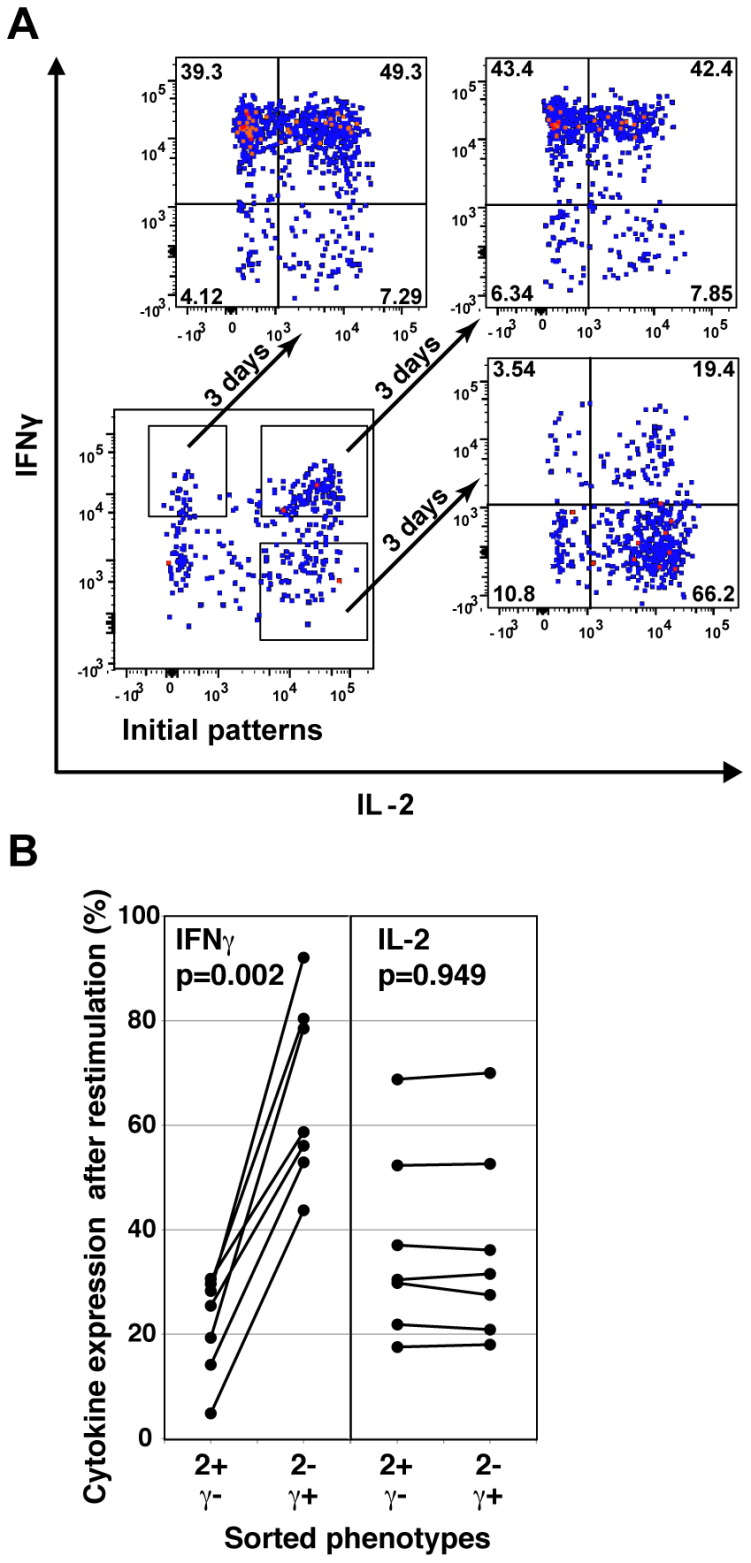
Stability and RNA expression differences between cytokine-secreting sub-populations of human SEB-stimulated CD4 T cells. Human PBMC were stimulated with SEB and the 2+γ+, 2+γ-, and 2-γ+ cells were isolated by cell sorting. (A) The three cell populations were cultured separately in neutral conditions for 3 days, then restimulated with PMA+Ionomycin and cytokine expression determined by ICS (representative experiment out of 3 independent experiments). (B) PBMC from seven subjects were stimulated with SEB and sorted into three populations as in A. Cytokine patterns in the sorted populations were determined by ICS after restimulation at 3 days.

To test the variability of cytokine patterns in human responses, we measured the *ex vivo* cytokine patterns expressed by CD4 T cells stimulated by the polyclonal activator SEB. Activated CD4+ CD45RA- CD69+ memory T cells were sorted into 2+γ+, 2-γ+, and 2+γ- sub-populations (Fig. S1) using the cytokine secretion assay, cultured separately in neutral conditions for three days, then restimulated using PMA/ionomycin. Cell populations initially expressing 2+γ+ or 2-γ+ patterns, contained similar proportions of IL-2+ and IL-2- cells after re-stimulation (Fig 1A, top two panels). This rapid convergence of the distinct sorted phenotypes into mixed IL-2-/+ populations was consistent with stochastic expression of IL-2 by the IFNγ+ cells. This instability of IL-2 expression was observed in additional subjects, with no significant difference between the levels of IL-2 produced on re-stimulation of the IL-2+ versus IL-2- sorted populations (Figure 1B).

In contrast to the rapid convergence of IL-2+ and IL-2- cells to similar mixed patterns, cells that were initially sorted as 2+γ- or 2+γ+ retained biased IFNγ expression patterns when re-stimulated, i.e. the majority of IFNγ+ cells expressed IFNγ on restimulation, whereas the majority of the IFNγ- cells did not (Fig. 1A, right two panels). In additional subjects, the proportion of cells producing IFNγ after restimulation of 2+γ+ cells was significantly higher than after restimulation of sorted 2+γ- cells (Figure 1B, p = 0.002). This suggested that the 2+γ- and 2+γ+ populations might include distinct, stable cell types.

Although IFNγ expression was more stable than that of IL-2, there were smaller numbers of IFNγ- to IFNγ+ and IFNγ+ to IFNγ- conversions, suggesting that there may also have been a smaller contribution of apparently stochastic expression of IFNγ. Thus many of the 2-γ+ and some of the 2+γ- cells may have been “lazy Th1” cells that did not express their full 2+γ+ potential during that particular stimulation cycle.

**Figure 2:**
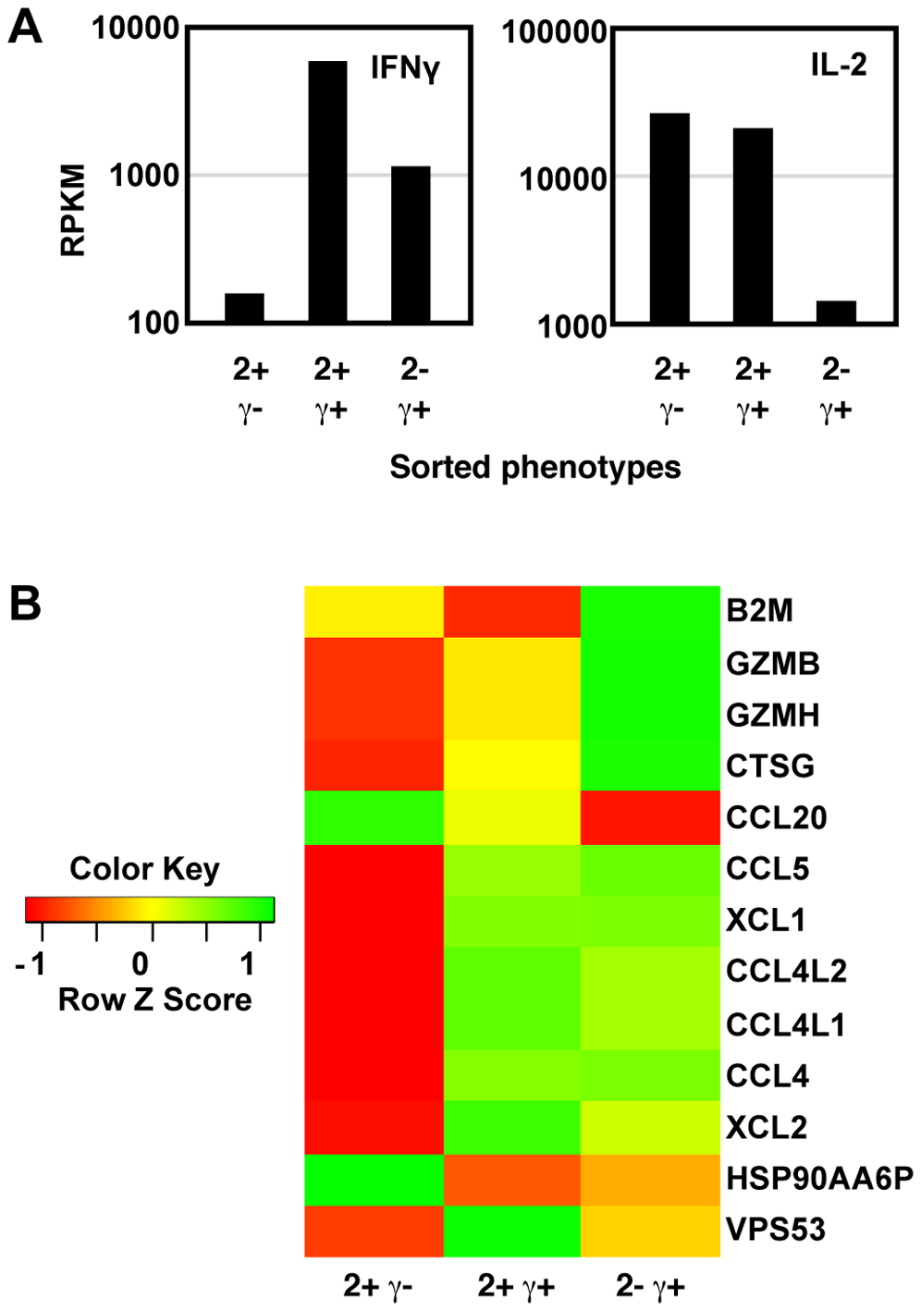
Differences in transcriptional patterns between IL-2+ IFNγ- cells and IFNγ+ cells. Human PBMC were stimulated with SEB and the 2+γ+, 2+γ-, and 2-γ+ cells were isolated by cell sorting. RNA was isolated immediately from sorted cells, and analyzed by RNA-seq. (A) The expression levels of IL-2 and IFNγ genes. (B) The heatmap shows the expression level of all the genes (in addition to IFNγ and IL-2) that were differentially expressed (p<0.001) between any two of the three cell populations.

If the IL-2+ and IL-2- populations, that rapidly re-oriented their IL-2 expression, were mainly due to random mechanisms then the gene expression profiles of these two sub-populations would be expected to be similar. In contrast, if the IFNγ+ and IFNγ- populations contained more stable phenotypes, gene expression may be more divergent. We therefore sequenced the mRNA of sorted 2+γ+, 2+γ- and 2-γ+ cell populations. Figure 2A shows that the abundance of mRNA for IFNγ and IL-2 matched the corresponding protein expression patterns defined by the sorting, thus confirming the validity of the RNAseq analysis of the sorted populations.

Gene expression levels were compared between the three cytokine-producing cell types, and genes ranked according to the significance of the differences between any pair of the three samples. Figure 2B shows the expression levels of the 13 genes (in addition to IL-2 and IFNγ) with the most significant differences among the three populations. Most of these genes were inducible T cell genes, including granzymes and chemokines. The 2+γ+ and 2-γ+ populations were more similar to each other, whereas the 2+γ- population showed larger differences with either of the other two populations.

### Cytokine pattern variability in the Th1-dominated human anti-influenza response

**Figure 3:**
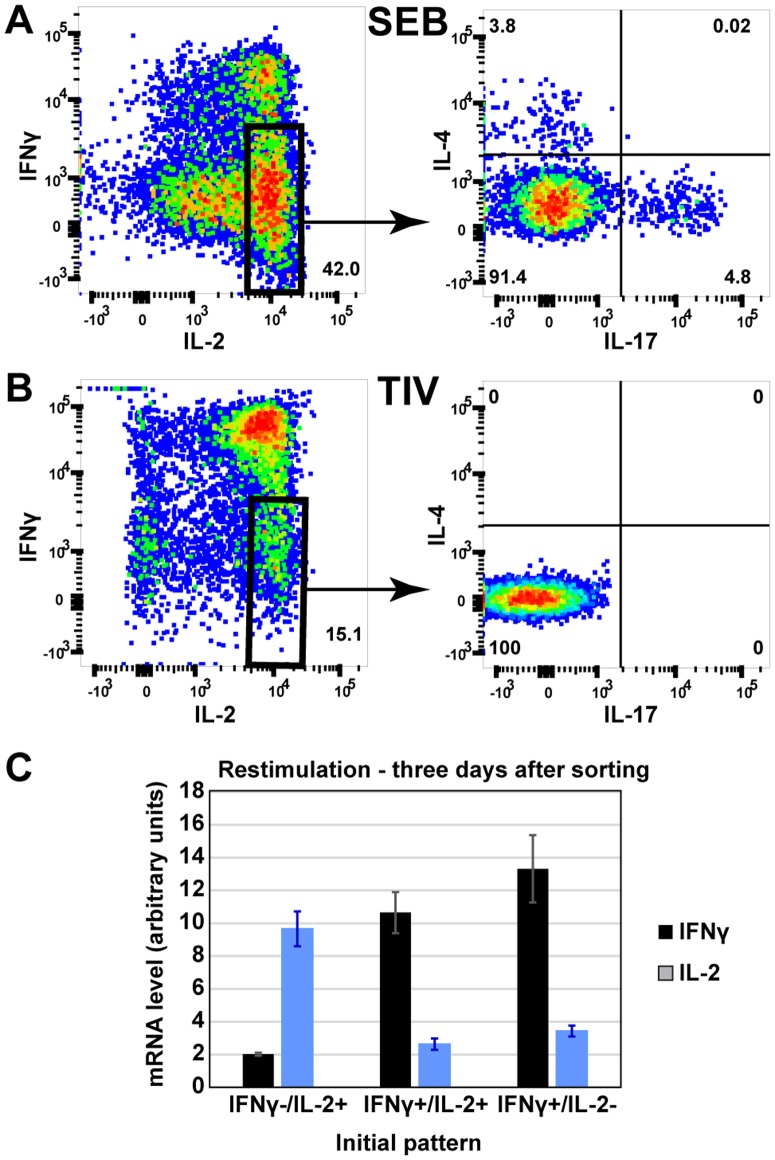
Stability of influenza-specific human CD4 T cells secreting different cytokine combinations. Human PBMC were stimulated with influenza TIV (A) or SEB (B), and expression of IL-2, IFNγ, IL-4 and IL17a was determined by ICS and flow cytometry. (C) Influenza specific human 2+γ+, 2+γ-, 2-γ+ CD4 T cells were sorted and cultured in neutral conditions for 3 days. The expression of IL-2 and IFNγ was measured by RT-PCR (n = 4). (Representative experiment out of 3 independent experiments).

Polyclonal stimulation by SEB activates many different T cell populations, for example the 2+γ- cells responding to SEB contain a small proportion of IL-4 and IL-17 producers (Fig. 3A) suggesting the presence of Th2-like and Th17-like cells (unlike mouse Th2 cells, human IL-4-producing T cells may also express IL-2). However, the human CD4 T cell response to influenza (Fig. 3B) is mainly a Th1 response, with no IL-4+ or IL-17+ cells detectable in the influenza specific 2+γ- population. We therefore tested IL-2 and IFNγ expression in the Th1-dominated influenza-specific response.

The stability of the influenza-specific IL-2 and IFNγ-producing cells was tested in similar experiments to the analysis of the SEB response (Figure S2). Due to the low numbers of antigen-specific T cells, real time PCR was used to measure IL-2 and IFNγ expression after sorting, culture and re-stimulation. Similar to cells responding to SEB, influenza-specific cells that were initially 2+γ+ or 2-γ+, converged to express similar amounts of IL-2 mRNA when restimulated (Fig. 3C). In contrast, the cells that were initially 2+γ- continued to produce mainly IL-2 but not IFNγ after culture in neutral conditions and restimulation, i.e. the bias in expression levels was maintained.

Although antigen concentration or costimulation could affect cytokine expression patterns [36], we found that anti-influenza responses that included a substantial proportion of 2+γ- cells still produced similar patterns when the antigen peptide dose was varied, or in the presence or absence of anti-CD28 antibodies (data not shown).

Thus in human influenza-specific memory CD4 T cells, the IFNγ+ cells may variably express IL-2, but 2+γ- cells may include cells with a distinct differentiation state. These cells might be memory CD4 cells that are not fully differentiated into effectors, such as the primed but uncommitted Thpp cells we have previously defined [21], [22].

### Reduced Tbet expression in IL-2+IFNγ- CD4 memory T cells

**Figure 4:**
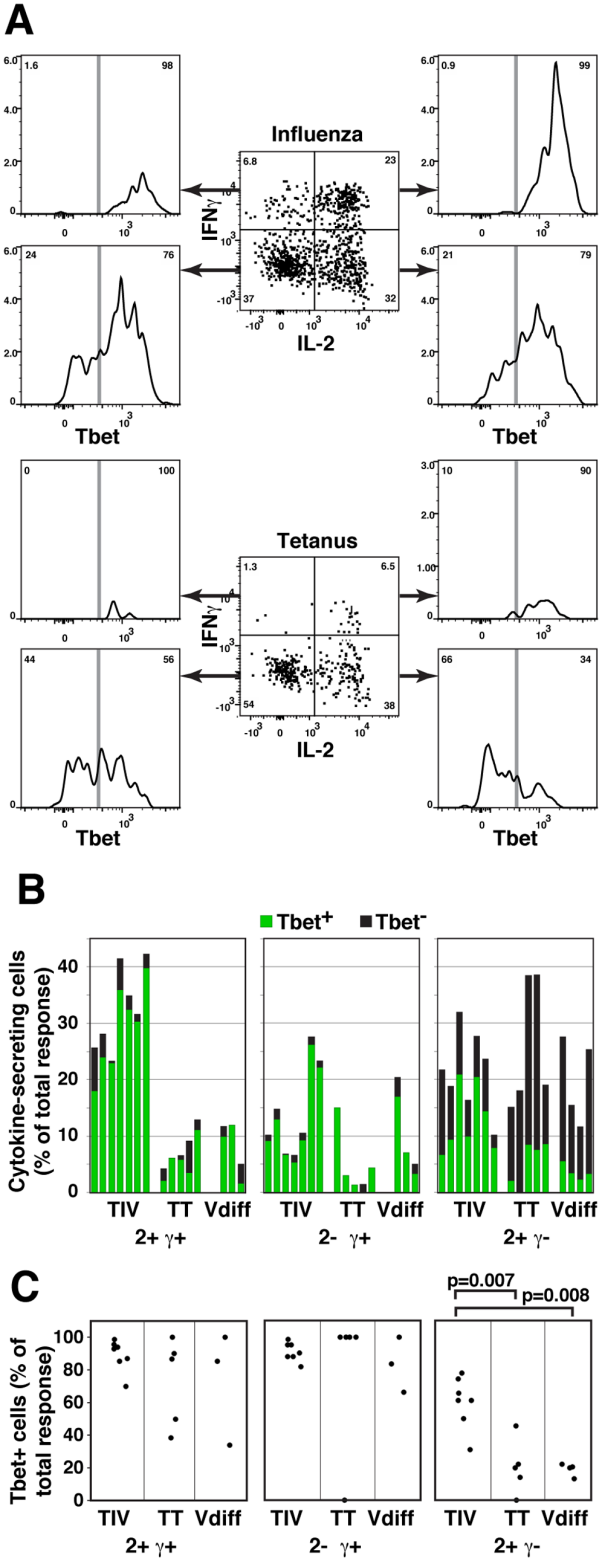
Tbet expression in influenza-specific 2+γ- and 2+γ+ cells. Human PMBC were stimulated with influenza vaccine (TIV), influenza A/California/7/2009 peptides (Vdiff) and tetanus peptides (TT), followed by ICS and analysis by flow cytometry. (A) Samples were gated on CD4+CD8-CD45RA-CD69+TNFα+ cells. IL-2 and IFNγ expression (center panels) and expression of Tbet in 2+γ+, 2+γ-, 2-γ+, and 2-γ- populations (outer panels) are shown in PBMC from one subject responding to TIV (upper panels) or TT (lower panels). (B) The proportion, relative to all responding cells, of Tbet+ and Tbet- cells is shown in 2+γ+, 2+γ-, and 2-γ+ populations responding to TIV, TT and Vdiff antigens. Each bar represents a different subject. (C) The proportion of Tbet+ cells is shown within each of the cytokine expression phenotypes.

If the 2+γ- cells contain significant numbers of uncommitted Thpp cells, this population should express lower levels of transcription factors associated with effector subset commitment, such as Tbet, GATA3 and RORγt. We compared Tbet expression in human influenza-specific CD4 T cells with Tbet levels in tetanus-specific cell populations, which contain uncommitted Thpp cells [22]. A very high proportion of influenza-specific 2+γ+ cells expressed Tbet (Fig. 4A), as expected for a clear Th1 population, whereas a majority of the tetanus-specific 2+γ- cells, presumed to be mostly Thpp cells, expressed low or absent levels of Tbet (Fig. 4A). Tetanus also stimulated a substantial population of Tbet- IL-2- IFNγ- TNFα+ cells, consistent with stochastic expression of IL-2 in the Thpp as well as the Th1 population. Although the proportion of 2+γ- cells was normally lower in influenza- than tetanus-specific responses, the influenza-specific 2+γ- cell population also included some Tbet^lo^ cells. Similar patterns of Tbet expression were observed in additional subjects (Figure 4B).

We previously found [24] that a high proportion of memory T cells specific for common, multiply-boosted influenza epitopes expressed IFNγ, whereas a higher proportion of 2+γ- cells were observed in responses against recent epitopes (influenza A/California/04/2009 peptides selected to have little cross-reactivity with previous influenza strains [24]). Figure 4B confirms that the percentages of IFNγ+ cells were lower in the CA/09 than TIV responses, and Figure 4C shows that a significantly higher proportion of the 2+γ- CA/09-specific cells were Tbet^lo^, in all four subjects.

**Figure 5:**
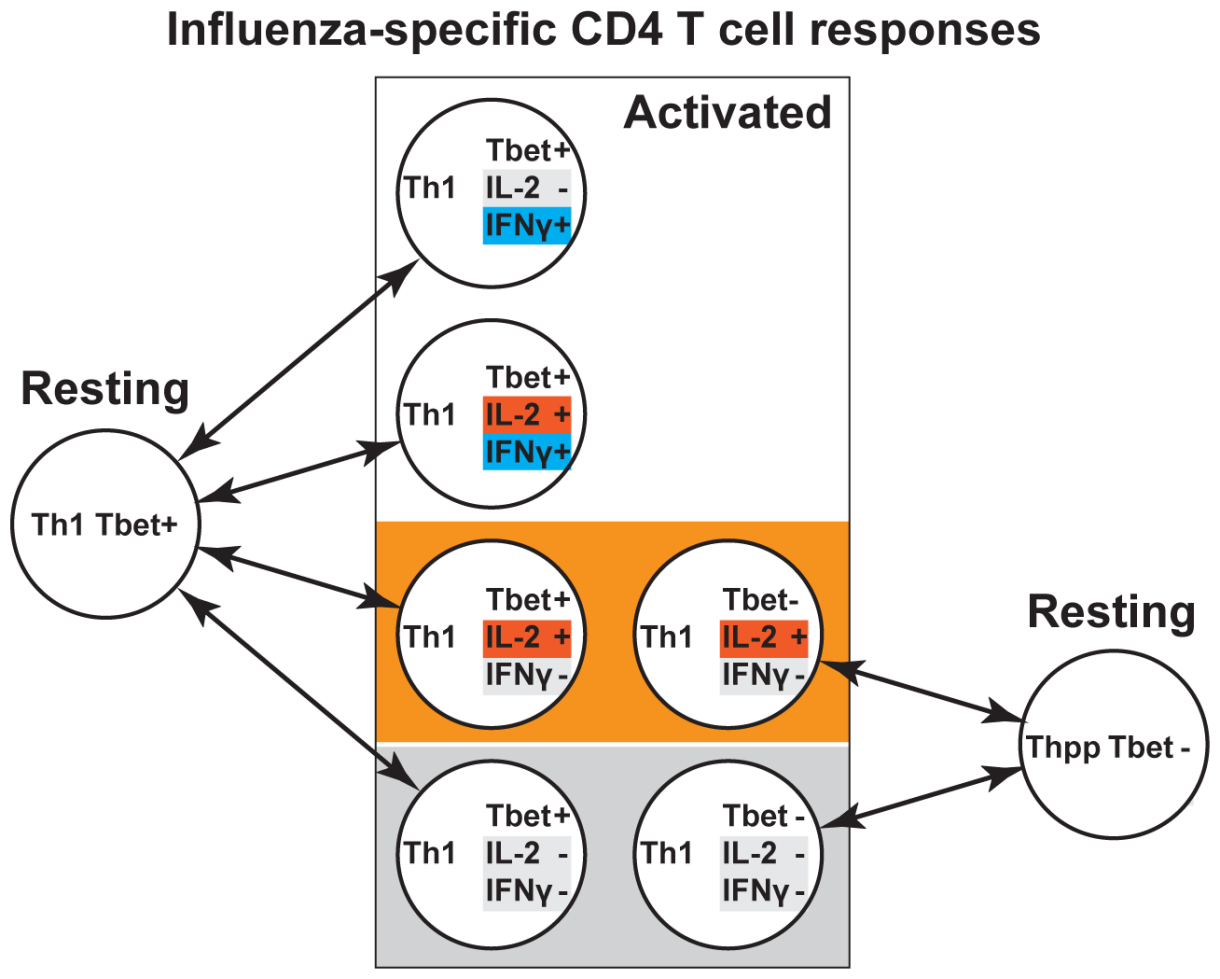
Model for Th1 and Thpp components in influenza-specific T cell memory. Stimulated Th1 cells (Tbet+) may express 2+γ+, 2+γ-, 2-γ+ or 2-γ- patterns after stimulation, whereas Thpp cells (Tbet-) may express only the 2+γ- or 2-γ- phenotypes. Thus the 2+γ- and 2+γ- cell populations may include both Thpp cells and Th1 cells.

These results are consistent with a model (Figure 5) in which the influenza and tetanus-specific memory CD4 T cell responses include differing proportions of at least two differentiation states, each capable of expressing multiple cytokine and transcription factor phenotypes – Th1 cells (Tbet+ TNFα+ IFNγ+/- IL-2+/-) and Thpp cells (Tbet- TNFα+ IFNγ- IL-2+/-).

### The influenza specific 2+γ- cell population contained uncommitted cells

In addition to their cytokine profile, one of the distinguishing characteristics of Thpp cells is the ability to further differentiate into effector cells, i.e. a single Thpp cell can give rise to either Th1 or Th2 cells [21], [22]. We therefore tested the re-differentiation potential of the influenza-specific 2+γ- cells.

**Figure 6:**
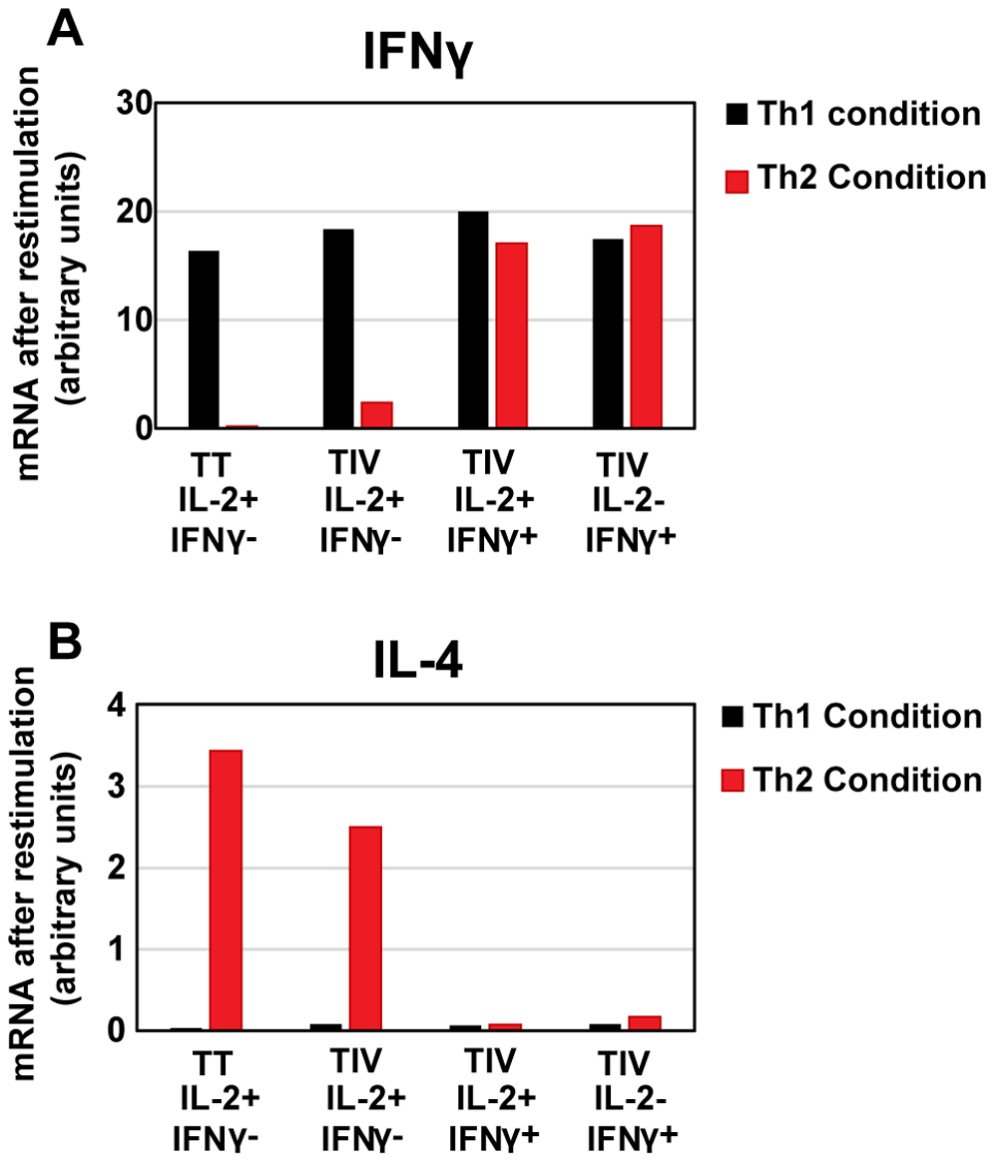
The influenza-specific 2+γ- cell population contains cells with differentiative potential. Influenza-specific 2+γ+, 2+γ-, and 2-γ+ cells and tetanus specific 2+γ- cells were sorted and cultured in Th1 conditions (A) or Th2 conditions (B) for 2 weeks. Then cultures were stimulated with PMA+Ionomycin for 6 hours and the expression of IFNγ (A) and IL-4 (B) determined by RT-PCR. (Representative experiment out of 3 independent experiments.).

Influenza-specific 2+γ- memory CD4 T cells were sorted using the cytokine secretion assay, and cultured in conditions that would induce naïve cells to differentiate into Th1 (IL-12 and anti-IL-4) or Th2 (IL-4 and anti-IFNγ). Influenza-specific IL-2+/- IFNγ+ cells were used as committed Th1 cell controls, and tetanus-specific 2+γ- cells as uncommitted Thpp cell controls [22]. After 2 weeks, the cells were re-stimulated with PMA+Ionomycin for 6 hours, and the expression levels of IFNγ and IL-4 mRNA were determined by real-time PCR. The two influenza-specific IFNγ+ populations expressed similarly high amounts of IFNγ on restimulation, whether cultured in Th1 or Th2 differentiation conditions (Fig. 6A). In contrast, the tetanus- and influenza-specific 2+γ- populations only developed high levels of IFNγ expression if cultured in Th1-inducing conditions, not in Th2 conditions.

As expected for committed Th1 cells, culturing the two IFNγ+ populations in Th2 conditions did not result in the differentiation of cells able to express IL-4 (Fig. 6B). However, the tetanus- and influenza-specific 2+γ- populations generated substantial amounts of IL-4 after culture in Th2 conditions. These results suggest that the 2-γ+ and 2+γ+ populations were committed Th1 cells, whereas both tetanus- and influenza-specific 2+γ- cells included substantial numbers of precursor cells that could become Th1- or Th2-like cells in the appropriate culture conditions.

## Discussion

The human CD4 memory T cell response to influenza can be explained by two types of diversity (Fig. 5). In this model, we propose that the majority of the anti-influenza response comprises Tbet+ Th1 cells that stochastically produce all combinations of IL-2 and IFNγ when restimulated *in vitro*. IL-2 expression appears to be reset rapidly, as sorted IL-2+ and IL-2- populations converge on similar mixtures of expression phenotypes within a few days. Some conversion between Tbet+ IFNγ+ and Tbet+ IFNγ- cells also occurs, but at a lower rate. In contrast to these apparently random effects, some of the 2+γ- cells appear to be more stable, and have the properties of the uncommitted Thpp cells described previously [22]. Only the 2+γ- population contains cells that can further differentiate into Th2- and Th1-like cells. Note that the stable or semi-stable differentiation phenotype (Th1, Thpp etc) cannot always be identified from the acute cytokine expression phenotype, as both Thpp and Th1 cells may express the 2+γ- or 2-γ- patterns. Tbet may be a more reliable marker of the Th1 phenotype, but Tbet can not be detected in live cells.

The 2+γ- Thpp cells share several characteristics with CD4+ central memory T cells (Tcm) [37] which were initially reported to produce IL-2 but not effector cytokines such as IFNγ or IL-4. Tcm and T effector memory cells (Tem) migrate preferentially to secondary lymphoid tissue and peripheral tissues, respectively [38], [39]. Tcm have better proliferation and survival ability [40] and express the homing marker CCR7, although CCR7 is not a completely reliable marker [41], [42]. Alternative definitions of Tcm have focused on CD62L expression, but these Tcm populations can include cells producing IFNγ [43], [44]. Thus Tcm populations (defined by homing markers) probably overlap substantially but not completely with Thpp populations (defined by cytokine expression and differentiation potential). Increased proliferation potential, considered a property of Tcm cells, might provide a further link between the Tcm and Thpp phenotypes, but this was not tested (e.g. by CFSE dilution) on the Thpp cells in our studies because of the low numbers of antigen-specific cells available.

The uncommitted Thpp cells are likely not equivalent to two other precursor populations that have been described: A CXCR5+ subset of Tcm could differentiate to Th1 or Th2 cells depending on the culture conditions [45], however, most of the 2+γ- cells in human PBMC responses to influenza or tetanus are CXCR5- [24]. Recently-described populations of CD95+ T memory stem cells (Tscm) have strong self-renewal potential and may be precursors of both Tcm and Tem [46]-[48]. However, Tscm express high levels of CD45RA [49], whereas Thpp cells express the same low levels of CD45RA as IFNγ-expressing Tem cells (J. Weaver, Y. Qi and T.R. Mosmann, data not shown).

Although 2-γ+ CD4 T cells could be terminally-differentiated, exhausted Th1 cells [26]–[28], sorted anti-influenza 2-γ+ cells, after restimulation, were able to express similar levels of IL-2 mRNA to the cells that were initially 2+γ+, suggesting that the 2-γ+ phenotype was more likely due to random expression patterns than exhausted Th1 cells.

In agreement with previous studies, our results are consistent with stochastic expression of the IL-2 and IFNγ genes in Th1 cells. The mechanism of cytokine stochastic expression may be related to monoallelic expression, which has been reported for several cytokines including IL-1α, IL-2, IL-4, IL-5, IL10, and IL-13 [17]–[19], [50]. Each allele of these genes can be expressed independently in individual cells, even though the two alleles share the same levels of the components of signaling pathways and transcription factors. Allelic expression could be regulated by differential accessibility of the two alleles [16]–[18], [50], [51], and it may also be possible that competitive binding of positive and negative regulators provides short-term stability of active or inactive states of each allele. In either case, accessibility or competitive binding could be changed during a subsequent unstimulated period, resulting in randomized allele expression on restimulation. Future studies could test whether the variable cytokine expression in the human anti-influenza response was related to allelic expression, by analyzing influenza-specific cells for the expression of individual SNP-marked alleles of IL-2 [52] in subjects pre-screened for SNP heterozygosity. For example, if the probability of each IL-2 allele being expressed in a single stimulation is 0.3, then the frequency of non-producers would be 0.7×0.7 = 0.49, and the frequency of single-allele expression would be 2×0.3×0.7 = 0.42, assuming that monoallelic expression was responsible for partial expression of IL-2.

Variable expression may contribute to the regulation of the quantity of cytokines produced by a T cell population, which is important because cytokines induce immune activation and pathogen destruction, yet excess amounts can severely damage host tissues. In addition to regulation of cytokine production by signal strength in individual cells [9], [36], the amount of cytokine could also be regulated at the population level, by controlling the number of cells that are activated to produce the cytokine. If the probability of cytokine gene expression is variable, then different proportions of the total population will activate 0, 1 or 2 alleles, contributing to a graded response of the population. This heterogeneity may provide a more graded response of cytokine quantities in different conditions [53], leading to more stable regulation of responses.

If communication between CD4 T cells and other cells, including DC, B cells, CD8 T cells and macrophages, operates preferentially at relatively short range [54]–[59] then variable expression of cytokines by individual T cells may also increase the variability of the phenotypes of these other effector cells. For example, IL-2 drives CD8 cells toward a memory cell phenotype, whereas IFNγ induces the CD8 cells to become effector cells [60]. Effector and memory cells are both essential, to provide current protection and future recall responses. CD4 T cells producing different IL-2 and IFNγ combinations will support the differentiation of both of these two cell types. Thus variability of CD4 T cell responses may contribute to the complexity of other effector populations.

Both tetanus and influenza vaccines also induce strong antibody responses, which probably depend mainly on Tfh cells [61], [62] rather than either Th1 or Thpp cells. Tfh cells are found mainly in lymphoid tissue but are rare in the circulation. Although Th1 cells can re-differentiate to Tfh-like cells [63], Tbet-deficient CD4 T cells have an increased ability to generate Tfh cells, both *in vivo* and *in vitro* [64]. Thus Tbet may suppress the differentiation of Tfh cells, and the Thpp cells (lacking Tbet- expression) might have an increased ability to differentiate into the Tfh cells that drive the antibody response that is important for protection against influenza. Such Thpp-derived Tfh might express fewer effector cytokines such as IFNγ or IL-17. The ratio between Th1, Thpp and Tfh differentiation may be regulated by the inflammatory environment during infection, but may also be affected by the individual properties of different naïve CD4 T cells [65], such as TCR affinity. In contrast to the potential of Thpp cells to contribute to Tfh populations, Th1 cells are more likely to contribute to cytotoxicity during influenza infection [4], as higher expression levels of Granzyme B were detected in IFNγ+ cells than IFNγ- cells (Fig 2B) and Tbet is required for expression of cytotoxic functions [66].

After proliferation and differentiation during an immune response, antigen-specific Thpp and Th1 cells will both be present at much higher frequencies than in the naïve CD4 T cell population. Thpp cells do not express high levels of effector cytokines, but have more proliferation potential [40]. Thpp may thus be equivalent to a stem cell-like model in which uncommitted central memory cells are more useful for future expansion and subsequent responses [67]. Thpp cells also provide increased flexibility of differentiation for future responses. In contrast, Th1 effector memory cells can produce effector cytokines immediately after infection and may be more useful for acute protection. Thus optimal and long-lasting protection against influenza infection may require memory responses that have an appropriate balance of the two cell types.

Thus the cytokine patterns expressed by CD4 T cells, even in the Th1-dominated response to influenza, can be determined by a combined effect of two mechanisms, short-term variability in cytokine expression, and semi-stable subset differentiation. Although the 2+γ- cytokine phenotype can be expressed by either Th1 or Thpp cells, the underlying differentiation state of the cell types is different, as revealed by selective expression of Tbet and other genes as well as future differentiation potential. Thus when measuring the T cell response to vaccines and infections, partial cytokine patterns have to be interpreted with caution. These results therefore have implications both for identification of the type of T cell response, and for the design of vaccines inducing the full depth of phenotypes.

## Supporting Information

Figure S1 **Gating strategy for human T cell sorting.** Sequential manual gating was performed to identify live lymphocytes (FSC-A/SSC-A); single cells (FSC-W/FSC-H and SSC-W/SSC-H); T cells (CD14/CD56/CD19); CD4 T cells (CD4/CD8); memory cells (CD45RA lo); and activated cells (CD154/TNFα). The final gates on IL-2 vs IFNγ were set apart slightly to enhance the purity of sorted cells. Proportions of 2+γ+ cells ranged from 30%–50% in different subjects, 2-γ+ cells from 5%–20%, and 2+γ- cells from 30%–60%.
(TIF)

Figure S2 **Gating strategy for human CD4 T cells responding to TIV, tetanus peptides or influenza peptides.** Sequential manual gating was performed to identify lymphocytes (FSC-A/SSC-A); single cells (FSC-W/FSC-H and SSC-W/SSC-H); T cells (CD14/CD3); CD4 T cells (CD4/CD8); live memory cells (Live-Dead/CD45RA); and activated cells (CD69/TNFα). Expression of IL-2, IFNγ and Tbet was then determined. The proportion of 2+γ+ cells ranged from 30%–70% in different subjects, 2−γ+ cells from 5%–30%, and 2+γ- cells from 20%–50%.
(TIF)

## References

[1] Allan W, Tabi Z, Cleary A, Doherty PC (1990) Cellular events in the lymph node and lung of mice with influenza. Consequences of depleting CD4+ T cells. J Immunol 144: 3980–3986. 1692070

[2] Wilkinson TM, Li CK, Chui CS, Huang AK, Perkins M, et al (2012) Preexisting influenza-specific CD4+ T cells correlate with disease protection against influenza challenge in humans. Nat Med 18: 274–280. 22286307 10.1038/nm.2612

[3] Mozdzanowska K, Maiese K, Gerhard W (2000) Th cell-deficient mice control influenza virus infection more effectively than Th- and B cell-deficient mice: evidence for a Th-independent contribution by B cells to virus clearance. J Immunol 164: 2635–2643. 10679103 10.4049/jimmunol.164.5.2635

[4] Brown DM, Lee S, Garcia-Hernandez Mde L, Swain SL (2012) Multifunctional CD4 cells expressing gamma interferon and perforin mediate protection against lethal influenza virus infection. J Virol 86: 6792–6803. 22491469 10.1128/JVI.07172-11 PMC3393557

[5] Topham DJ, Doherty PC (1998) Clearance of an influenza A virus by CD4+ T cells is inefficient in the absence of B cells. J Virol 72: 882–885. 9420305 10.1128/jvi.72.1.882-885.1998 PMC109454

[6] Teijaro JR, Verhoeven D, Page CA, Turner D, Farber DL (2010) Memory CD4 T cells direct protective responses to influenza virus in the lungs through helper-independent mechanisms. J Virol 84: 9217–9226. 20592069 10.1128/JVI.01069-10 PMC2937635

[7] Damjanovic D, Small CL, Jeyanathan M, McCormick S, Xing Z (2012) Immunopathology in influenza virus infection: uncoupling the friend from foe. Clin Immunol 144: 57–69. 22673491 10.1016/j.clim.2012.05.005

[8] Bucy RP, Panoskaltsis-Mortari A, Huang GQ, Li J, Karr L, et al (1994) Heterogeneity of single cell cytokine gene expression in clonal T cell populations. J Exp Med 180: 1251–1262. 7523568 10.1084/jem.180.4.1251 PMC2191707

[9] Darrah PA, Patel DT, De Luca PM, Lindsay RW, Davey DF, et al (2007) Multifunctional TH1 cells define a correlate of vaccine-mediated protection against Leishmania major. Nat Med 13: 843–850. 17558415 10.1038/nm1592

[10] Betts MR, Nason MC, West SM, De Rosa SC, Migueles SA, et al (2006) HIV nonprogressors preferentially maintain highly functional HIV-specific CD8+ T cells. Blood 107: 4781–4789. 16467198 10.1182/blood-2005-12-4818 PMC1895811

[11] Miller JD, van der Most RG, Akondy RS, Glidewell JT, Albott S, et al (2008) Human effector and memory CD8+ T cell responses to smallpox and yellow fever vaccines. Immunity 28: 710–722. 18468462 10.1016/j.immuni.2008.02.020

[12] Gaucher D, Therrien R, Kettaf N, Angermann BR, Boucher G, et al (2008) Yellow fever vaccine induces integrated multilineage and polyfunctional immune responses. J Exp Med 205: 3119–3131. 19047440 10.1084/jem.20082292 PMC2605227

[13] Akondy RS, Monson ND, Miller JD, Edupuganti S, Teuwen D, et al (2009) The yellow fever virus vaccine induces a broad and polyfunctional human memory CD8+ T cell response. J Immunol 183: 7919–7930. 19933869 10.4049/jimmunol.0803903 PMC3374958

[14] Precopio ML, Betts MR, Parrino J, Price DA, Gostick E, et al (2007) Immunization with vaccinia virus induces polyfunctional and phenotypically distinctive CD8(+) T cell responses. J Exp Med 204: 1405–1416. 17535971 10.1084/jem.20062363 PMC2118607

[15] Derrick SC, Yabe IM, Yang A, Morris SL (2011) Vaccine-induced anti-tuberculosis protective immunity in mice correlates with the magnitude and quality of multifunctional CD4 T cells. Vaccine. 10.1016/j.vaccine.2011.02.010 21338678

[16] Guo L, Hu-Li J, Paul WE (2004) Probabilistic regulation of IL-4 production in Th2 cells: accessibility at the Il4 locus. Immunity 20: 193–203. 14975241 10.1016/s1074-7613(04)00025-1

[17] Kelly BL, Locksley RM (2000) Coordinate regulation of the IL-4, IL-13, and IL-5 cytokine cluster in Th2 clones revealed by allelic expression patterns. J Immunol 165: 2982–2986. 10975806 10.4049/jimmunol.165.6.2982

[18] Paixao T, Carvalho TP, Calado DP, Carneiro J (2007) Quantitative insights into stochastic monoallelic expression of cytokine genes. Immunol Cell Biol 85: 315–322. 17438562 10.1038/sj.icb.7100057

[19] Hollander GA, Zuklys S, Morel C, Mizoguchi E, Mobisson K, et al (1998) Monoallelic expression of the interleukin-2 locus. Science 279: 2118–2121. 9516115 10.1126/science.279.5359.2118

[20] Jung T, Schauer U, Rieger C, Wagner K, Einsle K, et al (1995) Interleukin-4 and interleukin-5 are rarely co-expressed by human T cells. Eur J Immunol 25: 2413–2416. 7664804 10.1002/eji.1830250843

[21] Wang X, Mosmann T (2001) In vivo priming of CD4 T cells that produce interleukin (IL)-2 but not IL-4 or interferon (IFN)-gamma, and can subsequently differentiate into IL-4- or IFN-gamma-secreting cells. J Exp Med 194: 1069–1080. 11602637 10.1084/jem.194.8.1069 PMC2193514

[22] Divekar AA, Zaiss DM, Lee FE, Liu D, Topham DJ, et al (2006) Protein vaccines induce uncommitted IL-2-secreting human and mouse CD4 T cells, whereas infections induce more IFN-gamma-secreting cells. J Immunol 176: 1465–1473. 16424174 10.4049/jimmunol.176.3.1465

[23] Sad S, Mosmann TR (1994) Single IL-2-secreting precursor CD4 T cell can develop into either Th1 or Th2 cytokine secretion phenotype. J Immunol 153: 3514–3522. 7930573

[24] Weaver JM, Yang H, Roumanes D, Lee FE, Wu H, et al (2013) Increase in IFNgamma(-)IL-2(+) Cells in Recent Human CD4 T Cell Responses to 2009 Pandemic H1N1 Influenza. PLoS One 8: e57275. 23526940 10.1371/journal.pone.0057275 PMC3603952

[25] Mosmann TR, Kobie JJ, Lee FE, Quataert SA (2009) T helper cytokine patterns: defined subsets, random expression, and external modulation. Immunol Res. 10.1007/s12026-009-8098-5 19198763

[26] Han S, Asoyan A, Rabenstein H, Nakano N, Obst R (2010) Role of antigen persistence and dose for CD4+ T-cell exhaustion and recovery. Proc Natl Acad Sci U S A 107: 20453–20458. 21059929 10.1073/pnas.1008437107 PMC2996637

[27] Jeong YH, Jeon BY, Gu SH, Cho SN, Shin SJ, et al (2014) Differentiation of antigen-specific T cells with limited functional capacity during Mycobacterium tuberculosis infection. Infect Immun 82: 132–139. 24126533 10.1128/IAI.00480-13 PMC3911842

[28] Caserta S, Kleczkowska J, Mondino A, Zamoyska R (2010) Reduced functional avidity promotes central and effector memory CD4 T cell responses to tumor-associated antigens. J Immunol 185: 6545–6554. 21048115 10.4049/jimmunol.1001867

[29] Rangachari M, Zhu C, Sakuishi K, Xiao S, Karman J, et al (2012) Bat3 promotes T cell responses and autoimmunity by repressing Tim-3-mediated cell death and exhaustion. Nat Med 18: 1394–1400. 22863785 10.1038/nm.2871 PMC3491118

[30] Nakayama K, Nakamura H, Koga M, Koibuchi T, Fujii T, et al (2012) Imbalanced production of cytokines by T cells associates with the activation/exhaustion status of memory T cells in chronic HIV type 1 infection. AIDS Res Hum Retroviruses 28: 702–714. 21902582 10.1089/aid.2011.0073

[31] Diethelm-Okita BM, Raju R, Okita DK, Conti-Fine BM (1997) Epitope repertoire of human CD4+ T cells on tetanus toxin: identification of immunodominant sequence segments. J Infect Dis 175: 382–391. 9203659 10.1093/infdis/175.2.382

[32] Islam S, Kjallquist U, Moliner A, Zajac P, Fan JB, et al (2012) Highly multiplexed and strand-specific single-cell RNA 5′ end sequencing. Nat Protoc 7: 813–828. 22481528 10.1038/nprot.2012.022

[33] Wang L, Feng Z, Wang X, Wang X, Zhang X (2010) DEGseq: an R package for identifying differentially expressed genes from RNA-seq data. Bioinformatics 26: 136–138. 19855105 10.1093/bioinformatics/btp612

[34] Gazagne A, Claret E, Wijdenes J, Yssel H, Bousquet F, et al (2003) A Fluorospot assay to detect single T lymphocytes simultaneously producing multiple cytokines. J Immunol Methods 283: 91–98. 14659902 10.1016/j.jim.2003.08.013

[35] Rebhahn JA, Bishop C, Divekar AA, Jiminez-Garcia K, Kobie JJ, et al (2008) Automated analysis of two- and three-color fluorescent Elispot (Fluorospot) assays for cytokine secretion. Comput Methods Programs Biomed 92: 54–65. 18644656 10.1016/j.cmpb.2008.06.002 PMC4440339

[36] Itoh Y, Germain RN (1997) Single cell analysis reveals regulated hierarchical T cell antigen receptor signaling thresholds and intraclonal heterogeneity for individual cytokine responses of CD4+ T cells. J Exp Med 186: 757–766. 9271591 10.1084/jem.186.5.757 PMC2199012

[37] Sallusto F, Lenig D, Forster R, Lipp M, Lanzavecchia A (1999) Two subsets of memory T lymphocytes with distinct homing potentials and effector functions. Nature 401: 708–712. 10537110 10.1038/44385

[38] Masopust D, Vezys V, Marzo AL, Lefrancois L (2001) Preferential localization of effector memory cells in nonlymphoid tissue. Science 291: 2413–2417. 11264538 10.1126/science.1058867

[39] Reinhardt RL, Khoruts A, Merica R, Zell T, Jenkins MK (2001) Visualizing the generation of memory CD4 T cells in the whole body. Nature 410: 101–105. 11242050 10.1038/35065111

[40] Sallusto F, Geginat J, Lanzavecchia A (2004) Central memory and effector memory T cell subsets: function, generation, and maintenance. Annu Rev Immunol 22: 745–763. 15032595 10.1146/annurev.immunol.22.012703.104702

[41] Kim CH, Rott L, Kunkel EJ, Genovese MC, Andrew DP, et al (2001) Rules of chemokine receptor association with T cell polarization in vivo. J Clin Invest 108: 1331–1339. 11696578 10.1172/JCI13543 PMC209443

[42] Unsoeld H, Krautwald S, Voehringer D, Kunzendorf U, Pircher H (2002) Cutting edge: CCR7+ and CCR7- memory T cells do not differ in immediate effector cell function. J Immunol 169: 638–641. 12097363 10.4049/jimmunol.169.2.638

[43] Zaph C, Rook KA, Goldschmidt M, Mohrs M, Scott P, et al (2006) Persistence and function of central and effector memory CD4+ T cells following infection with a gastrointestinal helminth. J Immunol 177: 511–518. 16785548 10.4049/jimmunol.177.1.511 PMC1805702

[44] Stephens R, Langhorne J (2010) Effector memory Th1 CD4 T cells are maintained in a mouse model of chronic malaria. PLoS Pathog 6: e1001208. 21124875 10.1371/journal.ppat.1001208 PMC2991260

[45] Rivino L, Messi M, Jarrossay D, Lanzavecchia A, Sallusto F, et al (2004) Chemokine receptor expression identifies Pre-T helper (Th)1, Pre-Th2, and nonpolarized cells among human CD4+ central memory T cells. J Exp Med 200: 725–735. 15381728 10.1084/jem.20040774 PMC2211963

[46] Gattinoni L, Lugli E, Ji Y, Pos Z, Paulos CM, et al (2011) A human memory T cell subset with stem cell-like properties. Nat Med 17: 1290–1297. 21926977 10.1038/nm.2446 PMC3192229

[47] Zhang Y, Joe G, Hexner E, Zhu J, Emerson SG (2005) Host-reactive CD8+ memory stem cells in graft-versus-host disease. Nat Med 11: 1299–1305. 16288282 10.1038/nm1326

[48] Gattinoni L, Zhong XS, Palmer DC, Ji Y, Hinrichs CS, et al (2009) Wnt signaling arrests effector T cell differentiation and generates CD8+ memory stem cells. Nat Med 15: 808–813. 19525962 10.1038/nm.1982 PMC2707501

[49] Lugli E, Gattinoni L, Roberto A, Mavilio D, Price DA, et al (2013) Identification, isolation and in vitro expansion of human and nonhuman primate T stem cell memory cells. Nat Protoc 8: 33–42. 23222456 10.1038/nprot.2012.143 PMC6328292

[50] Bayley JP, van Rietschoten JG, Bakker AM, van Baarsen L, Kaijzel EL, et al (2003) Allele-specific expression of the IL-1 alpha gene in human CD4+ T cell clones. J Immunol 171: 2349–2353. 12928381 10.4049/jimmunol.171.5.2349

[51] Mariani L, Schulz EG, Lexberg MH, Helmstetter C, Radbruch A, et al (2010) Short-term memory in gene induction reveals the regulatory principle behind stochastic IL-4 expression. Mol Syst Biol 6: 359. 20393579 10.1038/msb.2010.13 PMC2872609

[52] Matesanz F, Delgado C, Fresno M, Alcina A (2000) Allelic selection of human IL-2 gene. Eur J Immunol 30: 3516–3521. 11093171 10.1002/1521-4141(2000012)30:12<3516::AID-IMMU3516>3.0.CO;2-S

[53] Hawkins ED, Turner ML, Dowling MR, van Gend C, Hodgkin PD (2007) A model of immune regulation as a consequence of randomized lymphocyte division and death times. Proc Natl Acad Sci U S A 104: 5032–5037. 17360353 10.1073/pnas.0700026104 PMC1821128

[54] de Goer de Herve MG, Dembele B, Vallee M, Herr F, Cariou A, et al (2010) Direct CD4 help provision following interaction of memory CD4 and CD8 T cells with distinct antigen-presenting dendritic cells. J Immunol 185: 1028–1036. 20562265 10.4049/jimmunol.0904209

[55] Kruman, II, Ramiya V, Bondada S (1996) A role for T cell CD4 in contact mediated T dependent B cell activation. Cell Immunol 173: 236–245. 8912882 10.1006/cimm.1996.0273

[56] Croft M, Swain SL (1991) B cell response to fresh and effector T helper cells. Role of cognate T-B interaction and the cytokines IL-2, IL-4, and IL-6. J Immunol 146: 4055–4064. 1828258

[57] Veldhoen M, Moncrieffe H, Hocking RJ, Atkins CJ, Stockinger B (2006) Modulation of dendritic cell function by naive and regulatory CD4+ T cells. J Immunol 176: 6202–6210. 16670330 10.4049/jimmunol.176.10.6202

[58] Sakai H, Okafuji I, Nishikomori R, Abe J, Izawa K, et al (2012) The CD40-CD40L axis and IFN-gamma play critical roles in Langhans giant cell formation. Int Immunol 24: 5–15. 22058328 10.1093/intimm/dxr088

[59] Bakocevic N, Worbs T, Davalos-Misslitz A, Forster R (2010) T cell-dendritic cell interaction dynamics during the induction of respiratory tolerance and immunity. J Immunol 184: 1317–1327. 20042584 10.4049/jimmunol.0902277

[60] Thaventhiran JE, Fearon DT, Gattinoni L (2013) Transcriptional regulation of effector and memory CD8+ T cell fates. Curr Opin Immunol 25: 321–328. 23747000 10.1016/j.coi.2013.05.010 PMC3766771

[61] Breitfeld D, Ohl L, Kremmer E, Ellwart J, Sallusto F, et al (2000) Follicular B helper T cells express CXC chemokine receptor 5, localize to B cell follicles, and support immunoglobulin production. J Exp Med 192: 1545–1552. 11104797 10.1084/jem.192.11.1545 PMC2193094

[62] Schaerli P, Willimann K, Lang AB, Lipp M, Loetscher P, et al (2000) CXC chemokine receptor 5 expression defines follicular homing T cells with B cell helper function. J Exp Med 192: 1553–1562. 11104798 10.1084/jem.192.11.1553 PMC2193097

[63] Oestreich KJ, Mohn SE, Weinmann AS (2012) Molecular mechanisms that control the expression and activity of Bcl-6 in T(H)1 cells to regulate flexibility with a T(FH)-like gene profile. Nat Immunol 13: 405–411. 22406686 10.1038/ni.2242 PMC3561768

[64] Nakayamada S, Kanno Y, Takahashi H, Jankovic D, Lu KT, et al (2011) Early Th1 cell differentiation is marked by a Tfh cell-like transition. Immunity 35: 919–931. 22195747 10.1016/j.immuni.2011.11.012 PMC3244883

[65] Tubo NJ, Pagan AJ, Taylor JJ, Nelson RW, Linehan JL, et al (2013) Single naive CD4+ T cells from a diverse repertoire produce different effector cell types during infection. Cell 153: 785–796. 23663778 10.1016/j.cell.2013.04.007 PMC3766899

[66] Hua L, Yao S, Pham D, Jiang L, Wright J, et al (2013) Cytokine-dependent induction of CD4+ T cells with cytotoxic potential during influenza virus infection. J Virol 87: 11884–11893. 23986597 10.1128/JVI.01461-13 PMC3807312

[67] Fearon DT, Carr JM, Telaranta A, Carrasco MJ, Thaventhiran JE (2006) The rationale for the IL-2-independent generation of the self-renewing central memory CD8+ T cells. Immunol Rev 211: 104–118. 16824121 10.1111/j.0105-2896.2006.00390.x

