# Supplementary figures and images for: Cytokine Diversity in the Th1-Dominated Human Anti-Influenza Response Caused by Variable Cytokine Expression by Th1 Cells, and a Minor Population of Uncommitted IL-2+IFNγ- Thpp Cells
Source: PLoS One. 2014 May 1;9(5):e95986. doi: 10.1371/journal.pone.0095986 (PMC4006810; doi:10.1371/journal.pone.0095986)

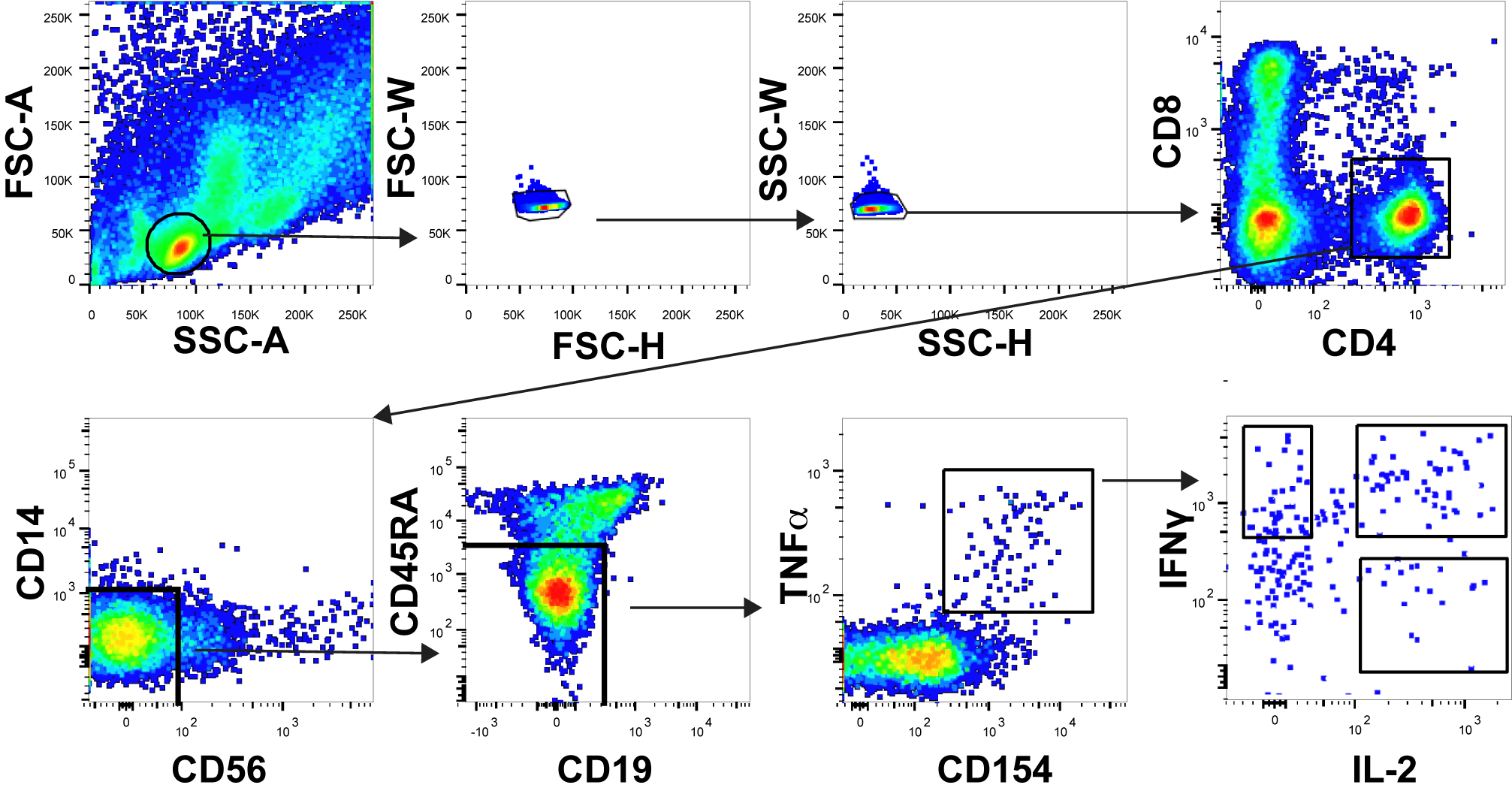

Supplement: Figure S1 — Gating strategy for human T cell sorting. Sequential manual gating was performed to identify live lymphocytes (FSC-A/SSC-A); single cells (FSC-W/FSC-H and SSC-W/SSC-H); T cells (CD14/CD56/CD19); CD4 T cells (CD4/CD8); memory cells (CD45RA lo); and activated cells (CD154/TNFα). The final gates on IL-2 vs IFNγ were set apart slightly to enhance the purity of sorted cells. Proportions of 2+γ+ cells ranged from 30%–50% in different subjects, 2-γ+ cells from 5%–20%, and 2+γ- cells from 30%–60%. (TIF) [file pone.0095986.s001.tif]

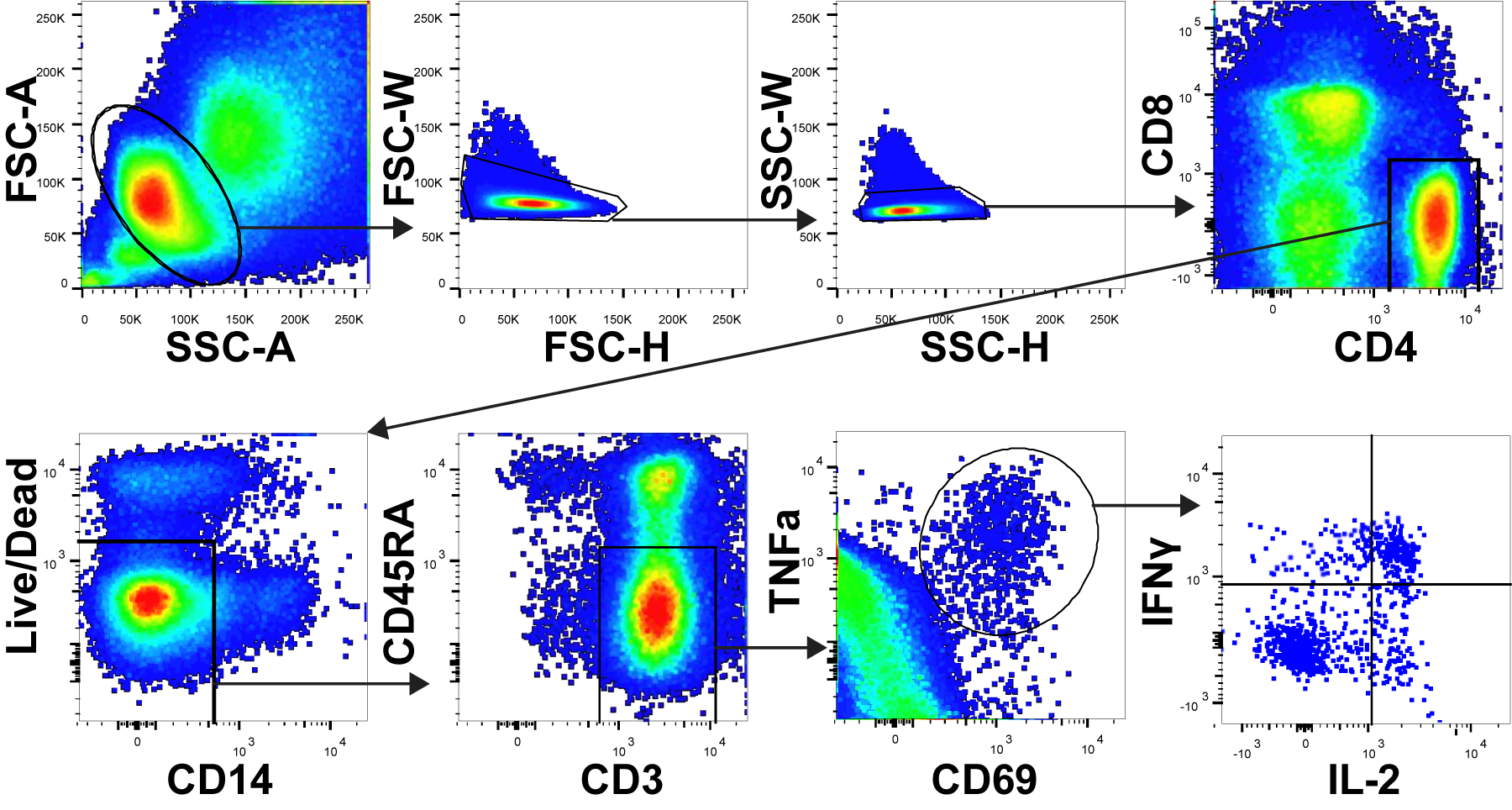

Supplement: Figure S2 — Gating strategy for human CD4 T cells responding to TIV, tetanus peptides or influenza peptides. Sequential manual gating was performed to identify lymphocytes (FSC-A/SSC-A); single cells (FSC-W/FSC-H and SSC-W/SSC-H); T cells (CD14/CD3); CD4 T cells (CD4/CD8); live memory cells (Live-Dead/CD45RA); and activated cells (CD69/TNFα). Expression of IL-2, IFNγ and Tbet was then determined. The proportion of 2+γ+ cells ranged from 30%–70% in different subjects, 2−γ+ cells from 5%–30%, and 2+γ- cells from 20%–50%. (TIF) [file pone.0095986.s002.tif]
